# Comparisons of GnRH Antagonist versus GnRH Agonist Protocol in Supposed Normal Ovarian Responders Undergoing IVF: A Systematic Review and Meta-Analysis

**DOI:** 10.1371/journal.pone.0106854

**Published:** 2014-09-12

**Authors:** Jin-song Xiao, Cun-mei Su, Xian-tao Zeng

**Affiliations:** 1 Reproductive Medicine Center, Taihe Hospital, Hubei University of medicine, Shiyan City, Hubei Province, China; 2 Reproduction Medicine and Treatment Center of Yunnan Province Population and Family Planning Science and Technology Institute, No. 150, Wuhua District, Kunming, Yunnan, China; 3 Evidence-Based Medicine Center of Hubei Medical College Affiliated Taihe Hospital, Shiyan City, HuBei, China; USA, United States of America

## Abstract

**Objective:**

To evaluate the effectiveness and safety of GnRH antagonist and GnRH agonist in supposed normal ovarian responders undergoing IVF.

**Methods:**

Data from 6 databases were retrieved for this study. The RCTs of GnRH agonist and GnRH antagonist use during IVF-EF therapy for patients with supposed normal ovarian response were included. A meta-analysis was performed with Revman 5.1software.

**Results:**

Twenty-three RCTs met the inclusion criteria. The number of stimulation days (mean difference (MD): −0.66, 95% confidence interval (CI): −1.04∼−0.27), Gn amount (MD: −2.92, 95% CI: −5.0∼−0.85), E2 values on the day of HCG (MD: −330.39, 95% CI: −510.51∼−150.26), Number of oocytes retrieved (MD: −1.33, 95% CI: −2.02∼−0.64), clinical pregnancy rate (odds ratio (OR): 0.87, 95% CI: 0.75−1.0), and ovarian hyperstimulation syndrome (OHSS) incidence (OR: 0.59, 95% CI: 0.42∼0.82) were significantly lower in GnRH antagonist protocol than GnRH agonist protocol. However, the endometrial thickness on the day of HCG (MD: −0.04, 95% CI: −0.23∼0.14), the ongoing pregnancy rate (OR: 0.87, 95% CI: 0.74∼1.03), live birth rate (OR: 0.89, 95% CI: 0.64∼1.24), miscarriage rate (OR: 1.17, 95% CI: 0.85∼1.61), and cycle cancellation rate (OR: 1.11, 95% CI: 0.90∼1.37) did not significantly differ between the 2 groups.

**Conclusions:**

During IVF treatment for patients with supposed normal responses, the incidence of OHSS were significantly lower, whereas the ongoing pregnancy and live birth rates were similar in the GnRH antagonist compared with the standard long GnRH agonist protocols.

## Introduction

It has been over 15 years since gonadotropin-releasing hormone (GnRH) antagonists were first applied in clinical practice in 1999. The debate regarding the efficacy and safety of GnRH antagonists and agonists for *in vitro* fertilisation - embryo transfer (IVF-ET)continues even today.

The specific binding of the GnRH antagonist to the GnRH pituitary receptor can suppress the surges of luteinising hormone (LH), feature a shorter ovarian stimulation time than the long protocol with a GnRH agonist, require a small amount of Gn, and have no flare-up effect. A systematic review of 5 randomised controlled trials (RCTs), conducted by Al-Inany in 2001 [1], showed that compared with the GnRH agonist long protocol, the GnRH antagonist fixed protocol showed a significantly reduced stimulation time and Gn amount, along with lower oocyte retrieved numbers and clinical pregnancy rates, whereas the incidence of severe ovarian hyperstimulation syndrome (OHSS) was not significantly different between the 2 treatment regimens. A systematic review of 27 RCTs, conducted by Al-Inany in 2006 [2], showed that the clinical pregnancy rate was significantly lower with GnRH antagonist treatment than with the GnRH agonist long protocol, while the differences in the ongoing pregnancy and live birth rates did not significantly differ between the 2 groups; however, the incidence of severe OHSS was significantly lower in the GnRH antagonist group. The live birth rate in a systematic review of 22 RCTs, conducted by Kolibianakis [3], was consistent with the findings reported by Al-Inany [2]. Another systematic review of 45 RCTs, conducted by Al-Inany in 2011 [4], reaffirmed the earlier results by the same author [2] with regard to the ongoing pregnancy and live birth rates and the incidence of severe OHSS. However, a review by Orvieto [5] stated that the ongoing pregnancy and live birth rates were significantly higher in the group treated according to the GnRH agonist long protocol compared to those treated with the GnRH antagonist and that the agonist protocol remained significantly better than the GnRH antagonist protocol. A meta-analysis by Pundir [6] showed that the incidence of moderate and severe OHSS was significantly lower in the GnRH antagonist group than in the GnRH agonist long protocol, while the incidence of severe OHSS was not significantly different.

Controlled ovarian hyperstimulation (COH) is an important component of IVF-ET technology. Different COH protocols would result in different ovarian responses in the same patient. The ovarian response to COH is an important factor that affects the pregnancy outcome, and different ovarian responses would produce different effects on pregnancy. Among the above-described systematic reviews, only the 2011 study by Al-Inany [4] conducted an analysis of all included patients, as well as of low-response and polycystic ovary syndrome (PCOS) subgroups. For all patients, the clinical pregnancy rate was significantly lower with the GnRH antagonist treatment than with the GnRH agonist long protocol, whereas the clinical pregnancy rates in the low-response and PCOS subgroups did not significantly differ, suggesting that the same COH protocol would cause different pregnancy outcomes in patients with different ovarian responses. Other studies [1]–[3], [5], [6] did not perform subgroup analyses based on the different ovarian responses of the patients. Those studies only compared the GnRH agonist and GnRH antagonist treatment regimens while ignoring the patients' characteristics and different pregnancy outcomes due to the different ovarian responses, and therefore, it is difficult to reach a consensus.

This dispute might be effectively resolved by evaluating the differences in the effects of the GnRH antagonist and GnRH agonist protocols based on the predicted ovarian responses of the patients. This study included RCTs of patients with supposed normal ovarian responses to systematically evaluate the effectiveness and safety of the GnRH antagonist and GnRH agonist long protocols for IVF.

## Methods

### Inclusion and exclusion criteria

The title and abstract of each study were read to filter out literature that obviously did not meet the inclusion criteria. Next, the full text of each study for possible inclusion was read to evaluate the included literature according to the inclusion and exclusion criteria.

All comparisons of the effectiveness and safety of GnRH agonists and GnRH antagonists for IVF in the context of RCTs were included, regardless of whether the blinding method was applied. The literature search was restricted to Chinese- and English-language articles.

Comparative studies of GnRH antagonists and GnRH agonists with other ovulation induction drugs, studies of GnRH antagonists without controls, and studies unrelated to the application effects of GnRH antagonists were excluded.

Studies of patients with a history of more than 3 IVF cycles, low or high ovarian response, PCOS, and severe endometriosis were excluded. Studies of GnRH antagonist in the context of minimal stimulation protocols and oocyte donation cycles were excluded.

The efficacy outcome measures included the number of stimulation days, given as the number of days of simulation; the Gn amount; the E2 value on the day of HCG; the number of oocytes retrieved; the endometrial thickness on the day of HCG; the clinical pregnancy rate, calculated as the number of pregnancies/the number of patients, for which clinical pregnancy was determined according to the detectable foetal heart beat in the intrauterine gestational sac by ultrasound; the ongoing pregnancy rate, which referred to pregnancies with over 12 weeks of gestation; and the live birth rate. The outcome measures of the safety included the incidence of OHSS, the miscarriage rate, and the cycle cancellation rate.

### Search strategy

Electronic databases, including PubMed (1997–2013), Cochrane Library (–2013), ProQuest Medical Library (PML; 1997–2013), Foreign Medical Journal Service (FMJS; 2000–2013), the Chinese Biomedical Database (CBM, 1979–2013), China National Knowledge Infrastructure (CNKI; 1994–2013), were all searched using the following keywords: “GnRH antagonist, GnRH-ant, GnRHA, GnRH agonist, GnRHa, IVF, Normal responders, Normoresponder”. The retrieval time was from the first publication of the journal until the end of December 2013. References included in the literature were also searched.

### Data extraction and quality assessment

The literature data extraction and quality assessment were independently completed and crosschecked by at least 2 trained qualified reviewers (XJS and SCM). If a disagreement occurred, a solution was achieved in a discussion with the third reviewer (ZXT).

The quality assessment of RCT complied with the assessing standards of risk of bias in the Cochrane Handbook for Systematic Reviews [7] (Version 5.1.0), including six aspects such as sequence generation, allocation concealment, blinding, incomplete outcome data, selective outcome reporting, and other biases.

### Statistical analysis

The statistical analysis was performed with Revman 5.1 software (Cochrane IMS; available at http://ims.cochrane.org/revman) using Version 5.1.0 of the Cochrane Handbook for Systematic Reviews [7] as a reference. Dichotomous variables were represented as odds ratios (OR), and continuous variables were expressed as mean differences (MD). The 95% confidence interval (CI) was used for all evaluation indicators, with the test level α = 0.05. Heterogeneity was evaluated by means of *I^2^* test, when *I* ^2^>50%, the included studies were considered to have large heterogeneity. The studies of non-statistical heterogeneity used the fixed effects model; the others of statistical heterogeneity used the subgroup analysis to find out the reason of the heterogeneity. If there was no clinical heterogeneity or methodological heterogeneity, the random effects model would be used. If there was significant clinical heterogeneity, the descriptive analysis was used. If needed, the sensitivity analysis was used to test stability of results. Publication bias was assessed with Begg's funnel plot carried out using Stata/SE, version 12.

## Results

### Screening Results

**Figure 1:**
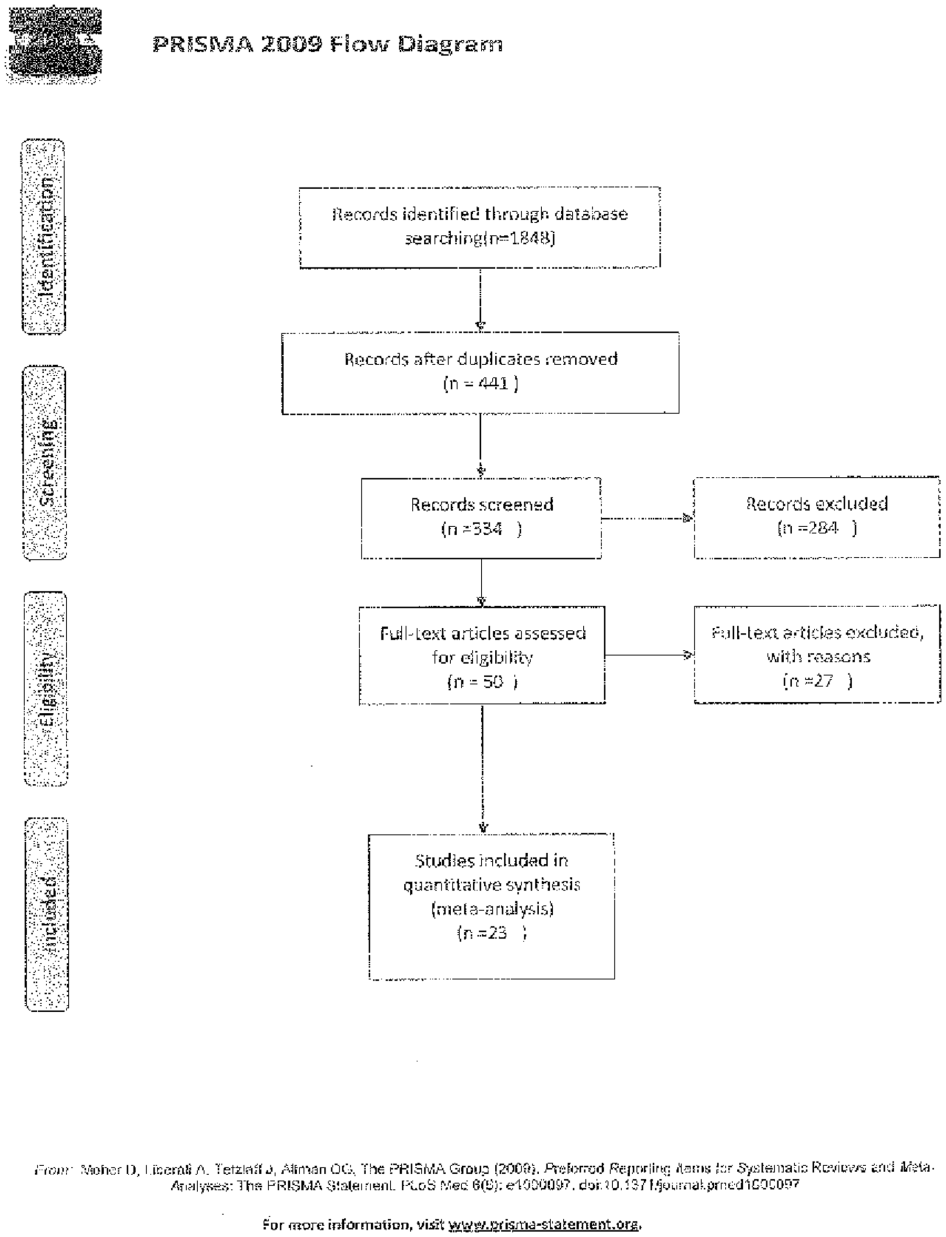
Prisma flow diagram.

A total of 1,848 studies were initially included in our study. After reading the titles and abstracts, 1,798 studies that did not meet the inclusion criteria or were duplicates were excluded. After reading the full text, 27 papers were excluded, and 23 published studies were ultimately included. The literature screening process and the results are shown in Figure 1. The basic characteristics of the included studies are shown in Table 1.

**Table 1:** Characteristics of included studies.

Study	Inclusion/Exclusion criteria	No. of patients		Age (y)		bFSH(IU/L)		BMI(kg/m^2^)		IVF Protocol	
		GnRH-ant	GnRH-a	GnRH-ant	GnRH-a	GnRH-ant	GnRH-a	GnRH-ant	GnRH-a	GnRH-ant	GnRH-a
Albano *et al*.,2000 [8]	Normal menstrual cycle, FSH levels <10 IU/L, and previous IVF cycle <3.	198	95	31.9±3.7	31.6±3.8	N/A	N/A	N/A	N/A	Multiple dose (cetrorelix)	Long, multiple dose (buserelin)
European orgalutran, 2000 [9]	Age<39, with normal menstrual cycles, BMI18–29 kg/m^2^	463	238	31.9±3.6	31.9±3.8	N/A	N/A	23.0±2.9	23.0±2.7	Multiple dose (ganirelix)	Long, multiple dose (buserelin)
Olivennes *et al*., 2000 [10]	Normal menstrual cycle, FSH levels <10 IU/L, normal uterus, previous IVF cycle <3.	126	43	31.4±3.7	31.8±3.8	6.3±2.0	6.3±1.9	N/A	N/A	Single dose (cetrorelix)	Long, single dose (triptorelin)
Eroupean-Middle East, 2001 [11]	Age<39, with normal menstrual cycles, BMI18–29 kg/m^2^	226	108	N/A	N/A	N/A	N/A	N/A	N/A	Multiple dose (ganirelix)	Long, single dose (triptorelin)
North American,2001 [12]	Age <39, with normal menstrual cycles	198	99	33.0±3.4	32.8±4.0	N/A	N/A	23.0±3.0	23.0±3.0	Multiple dose (ganirelix)	Long, multiple dose (leuprorelin)
Hohmann *et al*.,2003 [13]	Previous IVF cycle <3, no previous IVF cycle with a poor response or OHSS.	48	45	33(26–38)*	33(25–39)*	6.3(2–16)*	5.5(1.0–10.8*	24.2 (19.7–28.4)*	23.0(19.6–28.1)*	Multiple dose (cetrorelix)	Long, single dose (triptorelin)
Check *et al*., 2004 [14]	No description	24	30	38.0±5.0	32.7±3.8	N/A	N/A	N/A	N/A	Multiple dose (ganirelix)	Long, multiple dose (leuprorelin)
Lee *et al*.,2004 [15]	Regular menstruation cycles; no history of poor ovarian response or reserve.	20	20	31.7±3.8	32.8±4.4	6.68±1.75	6.33±1.41	21.76±3.63	20.98±2.45	Multiple dose (cetrorelix)	Long, multiple dose (buserelin)
Loutradis *et al*., 2004 [16]	No low response in a previous treatment cycle, regular menstrual cycles.	58	58	35.8±4.9	34.9±4.7	6.3±1.5	6.0±1.3	N/A	N/A	Multiple dose (cetrorelix)	Long, multiple dose (triptorelin)
Sauer *et al*.,2004 [17]	Regular menstrual cycles, both ovaries present.	93	98	32.6±4.0		N/A	N/A	24.2±4.5		Single dose (cetrorelix)	Long, multiple dose (leuprorelin)
Barmat *et al*., 2005 [18]	AFC>5 with a menstrual cycle, and failed IVF or IVF/ICSI cycle<1. Patients were excluded from the study if they had a history of previous poor response.	39	41	32.4±0.4	32.2±0.4	6.6±0.3	6.3±0.3	24.7±0.6	23.8±0.5	Multiple dose (ganirelix)	Long, multiple dose (leuprorelin)
Xavier *et al*., 2005 [19]	previous IVF cycle <3.	53	59	31.8±3.0	30.6±2.8	6.4±1.2	6.3±1.0	N/A	N/A	Multiple dose (cetrorelix)	Long, multiple dose (buserelin)
Friedler *et al*.,2006 [20]	The patients were excluded from the study if they had previous IVF or ICSI,	56	40	28.36±3.1	28.71±2.8	5.54±1.1	5.77±1.2	27.54±4.3	28.1±3.4	Multiple dose (ganirelix)	Long, multiple dose (buserelin)
Serafini *et al*.,2006 [21]	The presence of two functional ovaries; previous IVF cycle <3; no history of low ovarian response in previous IVF/ICSI treatment.	93	98	34.4±0.4	33.4±0.3	8.0±2.3	8.8±2.7	≤25	≤25	Multiple dose (cetrorelix)	Long, multiple dose (leuprorelin)
Rombauts *et al*., 2006 [22]	Exclusion criteria included endocrineabnormalities(e.g.PCOS),unsuccessful COS cycles>3, low or no ovarian response.	110	111	32.1±3.7	32.2±4.0	N/A	N/A	23.4±3.0	24.2±3.6	Multiple dose (ganirelix)	Long, multiple dose (Nafarelin)
Baart *et al*., 2007 [23]	Regular menstrual cycles, BMI between 19 and 29 kg/m^2^	56	40	33.2(22–37)*	34.1(28–37)*	7.6(5.5–18.4*	8.1(4.4–13.8*	N/A	N/A	Multiple dose (orgalutran)	Long, multiple dose (triptorelin)
Hsieh *et al*., 2008 [24]	Age 18–39 years; and body weight of 40–70 kg.	86	58	33.9±4.4	30.9±2.5	4.0±1.8	3.8±1.4	20.6±1.4	20.7±2.1	Multiple dose (cetrorelix)	Long, multiple dose (leuprorelin)
Moraloglu *et al*.,2008 [25]	Patients were excluded from the study;a history of previous poor response, previous IVF cycles>3, and PCOS.	45	48	30.91±5.52	30.25±4.94	6.63±1.33	6.32±1.77	29.36±4.45	26.58±3.32	Multiple dose (cetrorelix)	Long, multiple dose (leuprorelin)
Depalo *et al*.,2009 [26]	Absence of uterine or ovarian abnormalities or severe endometriosis or PCOS, previous IVF cycles≤3.	67	69	34.4±4	34±3.9	6.4±2.4	5.7±2	23.7±4.1	22.7±3.4	Multiple dose (cetrorelix)	Long, multiple dose (triptorelin)
Ye *et al*., 2009 [27]	Previous IVF cycles <3, and no previous poor response to ovarian stimulation; normal ovulatory cycles.	109	111	30.3±2.8	30.2±2.8	6.2±1.6	6.5±1.3	20.7±1.9	21.0±1.8	Multiple dose (cetrorelix)	Long, multiple dose (triptorelin)
Firouzabadi *et al*.,2010 [28]	The first cycle of the ART, age <35 years, and bFSH <10 IU/L.	110	100	28.36±3.1	28.71±2.8	5.54±1.1	5.77±1.2	27.54±4.3	28.1±3.4	Multiple dose (ganirelix)	Long, multiple dose (buserelin)
Qiao *et al*.,2012 [29]	Aged ≥18 and ≤35 years, with BMI18–29 kg/m^2^, a normal menstrual cycle.	113	120	29.3±2.8	29.1±3.0	N/A	N/A	21.3±2.2	21.3±2.4	Multiple dose (ganirelix)	Long, multiple dose (triptorelin)
Papanikolaou *et al*. 2012 [30]	Age<39 years; FSH<12 mIU/ml, previous IVF cycles <3.	96	94	32.2±0.3	32.8±0.3	N/A	N/A	N/A	N/A	Multiple dose(ganirelix/cetrorelix)	Long, multiple dose (buserelin)

bFSH = basal follicle stimulating hormone; BMI = body mass index; IVF = in vitro fertilization; AFC = antral follicle count; ICSI = intracytoplasmic sperm injection; COS = controlled ovarian stimulation; OHSS = ovarian hyperstimulation syndrome; PCOS = polycystic ovary syndrome; ART = assisted reproductive technology; N/A =  Not available.

*median.

### Quality Assessment

A total of 23 (3,961 cases) RCTs that compared GnRH antagonist and GnRH agonist long protocol treatments were included, The quality assessment of RCT complied with the assessing standards of risk of bias in the Cochrane Handbook for Systematic Reviews V5.1.0, including sequence generation, allocation concealment, blinding, incomplete outcome data, selective outcome reporting, and the baseline consistency., as shown in Figure S1 in File S1.

### Outcome Measures of the Effectiveness

#### Number of stimulation days

This outcome measure was included in 16 studies [8]–[12], [15]–[20], [22], [25]–[27], [30] (3,118 cases), and heterogeneity was observed among various trials (*P*<0.0001, *I* ^2^ = 91%). Therefore, a random-effect model was used for the meta-analysis. The results showed that the number of stimulation days was significantly less in the GnRH antagonist group than in the GnRH agonist group; this difference was statistically significant (MD: −0.66, 95%CI: −1.04∼−0.27; *P* = 0.008; Figure S2 in File S1).

#### Gn amount

This outcome measure was included in 15 studies [8], [10], [15]–[17], [19]–[28] (2,086 cases), and heterogeneity was observed among various trials (*P*<0.00001, *I* ^2^ = 96%). Therefore, a random-effect model was used for the meta-analysis. The results showed that the Gn amount was significantly less in the GnRH antagonist group than in the GnRH agonist group; this difference was statistically significant (MD: −2.92,95%CI:−5.0∼−0.85; *P* = 0.006; Figure S3 in File S1).

#### Endometrial thickness on the day of HCG

This outcome measure was evaluated in 5 studies [15], [19], [20], [27], [28] (655 cases), and no statistical heterogeneity was observed among various trials (*P* = 0.79, *I* ^2^ = 0%). Therefore, a fixed-effect model was used for the meta-analysis. The results showed that there was no statistically significant difference in endometrial thickness on the day of HCG between the GnRH antagonist group and the GnRH agonist group (MD: −0.04,95%CI:−0.23∼0.14; *P* = 0.64; Figure S4 in File S1).

#### E2 value on the day of HCG

This outcome measure was evaluated in 15 studies [8]–[12], [15]–[19], [21], [24]–[26], [28] (2,807 cases; the unified international standard unit pg/ml was adopted, with a conversion factor of 3.67), and heterogeneity was observed among various trials (*P*<0.00001, *I* ^2^ = 96%). Therefore, a random-effect model was used for the meta-analysis. The results showed that the E2 value on the day of HCG was lower in the GnRH antagonist group than in the GnRH agonist group, and this difference was statistically significant (MD: −330.39,95%CI: −510.51∼−150.26; *P* = 0.0003; Figure S5 in File S1).

#### Number of oocytes retrieved

This outcome measure was included in 20 studies [8]–[12], [14]–[16], [19]–[30] (4,328 cases), and heterogeneity was observed among various trials (*P*<0.00001, *I* ^2^ = 88%). Therefore, a random-effect model was used for the meta-analysis. The results showed that the number of oocytes retrieved was lower in the GnRH antagonist group than in the GnRH agonist group, and this difference was statistically significant (MD: −1.33,95%CI: −2.02∼−0.64; *P* = 0.0001; Figure S6 in File S1).

#### Clinical Pregnancy rate

**Figure 2:**
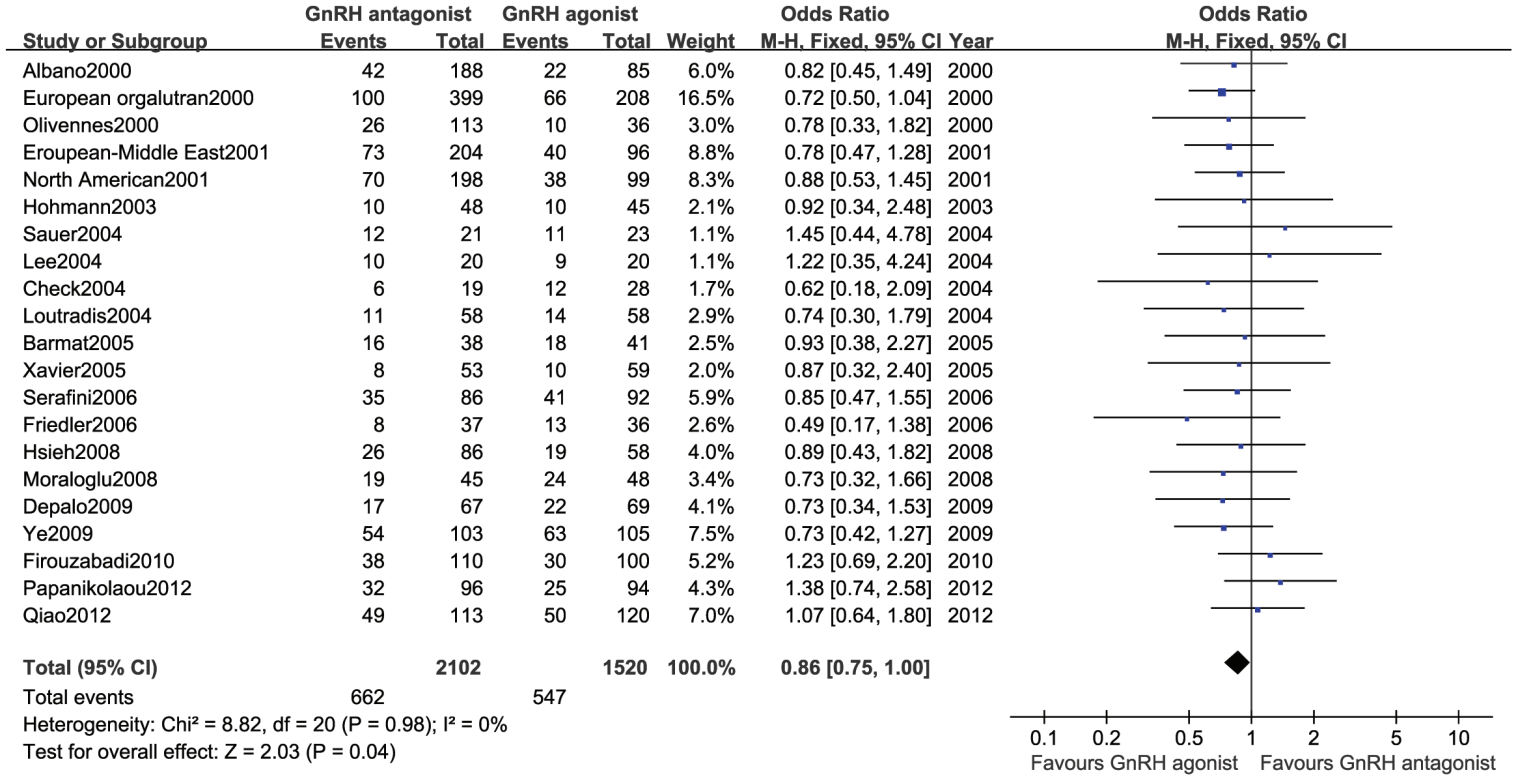
Forest plot of the comparison of the GnRH antagonist group versus the GnRH agonist group for clinical pregnancy rate.

This outcome measure was included in 21 studies [8]–[21], [24]–[30] (3,622 cases), and no statistical heterogeneity was observed among various trials (*P* = 0.98, *I* ^2^ = 0%). Therefore, a fixed-effect model was used for the meta-analysis. The results showed that the clinical pregnancy rate was lower in the GnRH antagonist group than in the GnRH agonist group, and this difference was statistically significant (OR:0.86,95%CI:0.75∼1.00; *P* = 0.04; Figure 2).

#### Ongoing pregnancy rate

**Figure 3:**
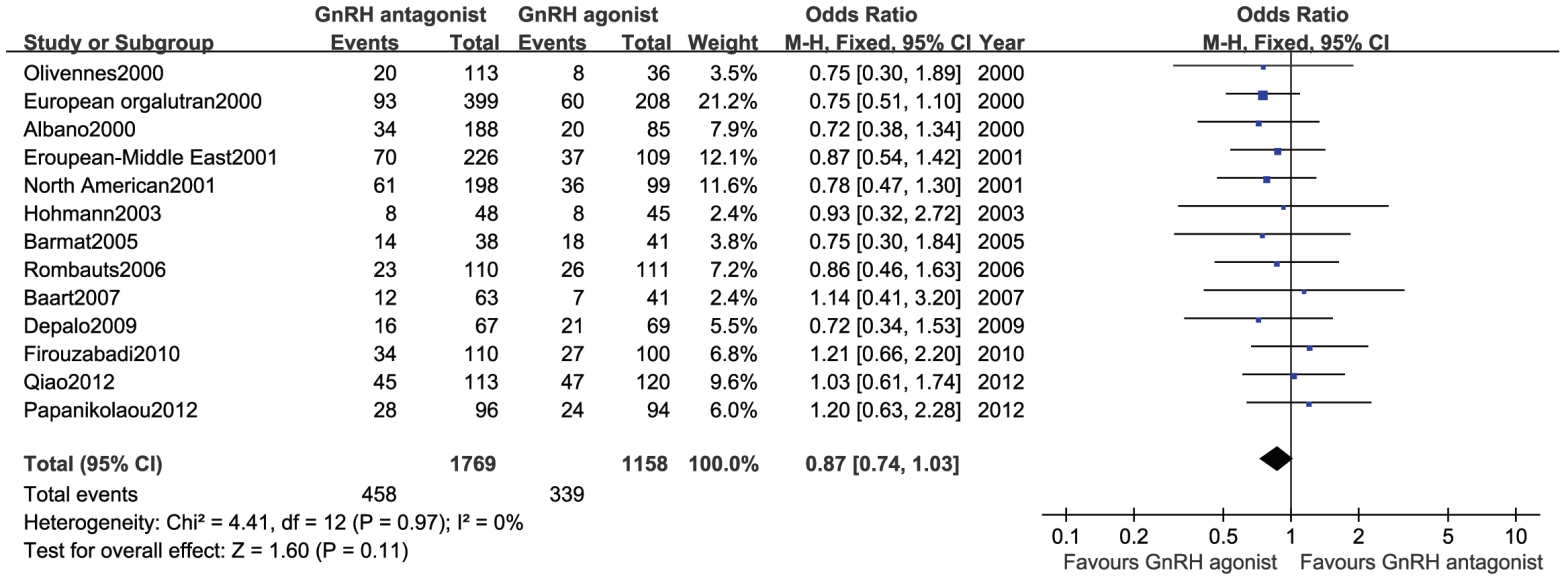
Forest plot of the comparison of the GnRH antagonist group versus the GnRH agonist group for ongoing pregnancy rate.

This outcome measure was included in 14 studies [8]–[13], [18], [22], [23], [26], [28]–[30] (2,927 cases), and no statistical heterogeneity was observed among various trials (*P* = 0.97, *I* ^2^ = 0%). Therefore, a fixed-effect model was used for the meta-analysis. The results showed that there was no statistically significant difference in the ongoing pregnancy rate between the GnRH antagonist group and the GnRH agonist group (OR:0.87,95%CI:0.74∼1.03; *P* = 0.11; Figure 3).

#### Live birth rate

**Figure 4:**
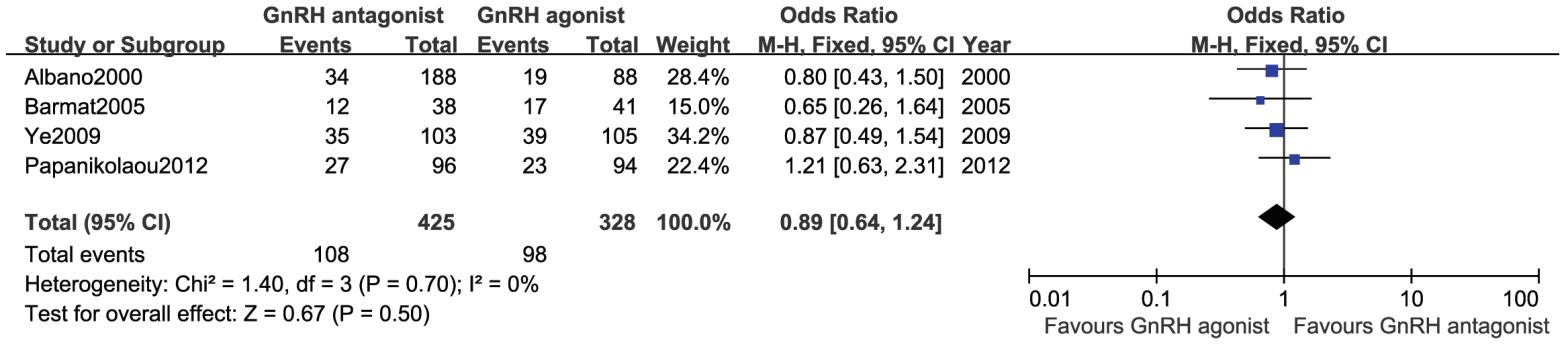
Forest plot of the comparison of the GnRH antagonist group versus the GnRH agonist group for live birth rate.

This outcome measure was included in 4 studies [8], [18], [27], [30] (753 cases), and no statistical heterogeneity was observed among various trials (*P* = 0.84, *I* ^2^ = 0%). Therefore, a fixed-effect model was used for the meta-analysis. The results showed that there was no statistically significant difference in the live birth rate between the GnRH antagonist group and the GnRH agonist group (OR:0.89,95%CI:0.64∼1.24; *P* = 0.50; Figure 4).

### Outcome Measures of the Safety

#### Incidence of OHSS

**Figure 5:**
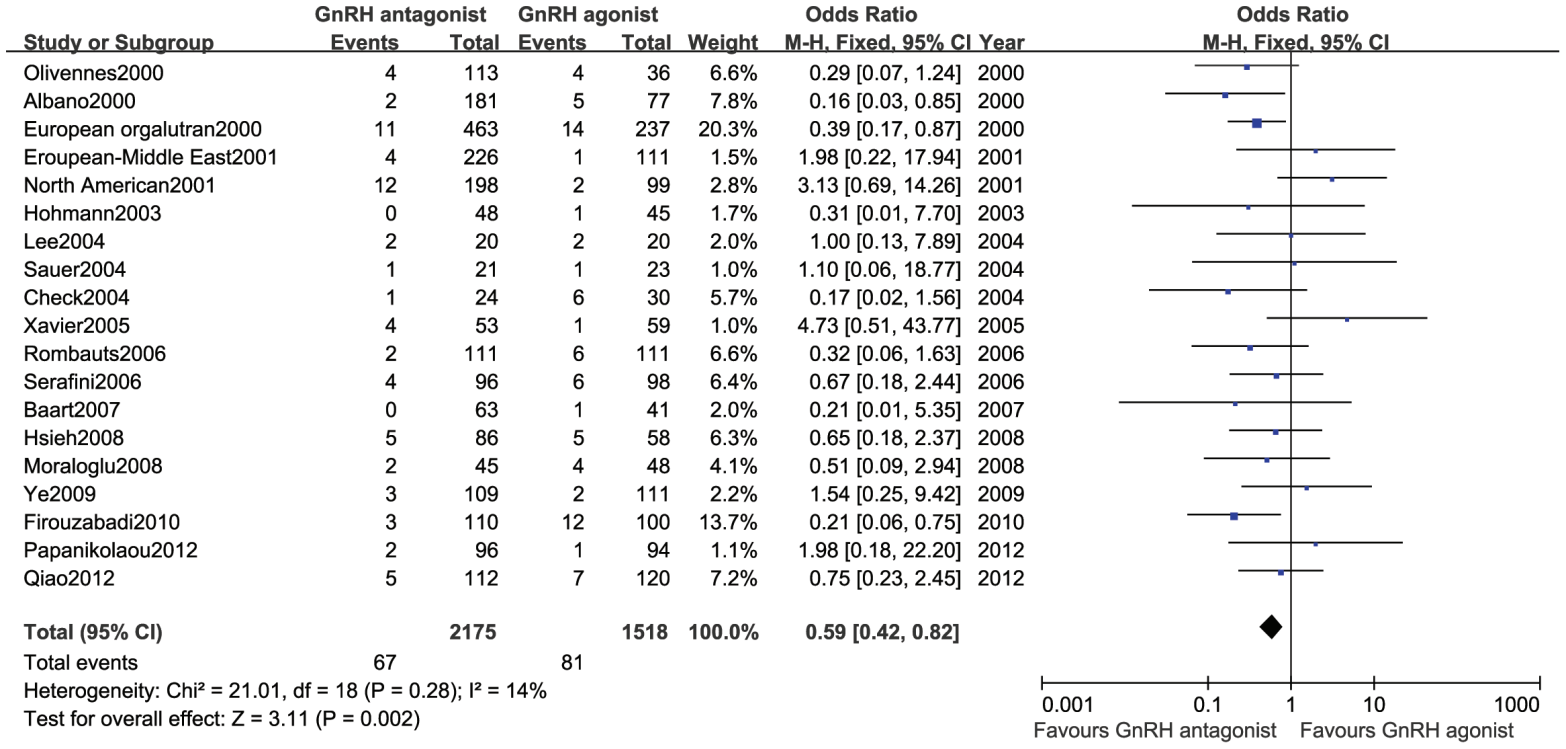
Forest plot of the comparison of the GnRH antagonist group versus the GnRH agonist group for incidence of OHSS.

This outcome measure was included in 20 studies [8]–[15], [17], [19], [21]–[25], [27]–[30] (3,693 cases), and no statistical heterogeneity was observed among various trials (*P* = 0.28, *I* ^2^ = 14%). Therefore, a fixed-effect model was used for the meta-analysis. The results showed that the incidence of OHSS was lower in the GnRH antagonist group than in the GnRH agonist group, and this difference was statistically significant (OR:0.59,95%CI:0.42∼0.82; *P* = 0.002; Figure 5).

#### Miscarriage rate

This outcome measure was included in 17 studies [8]–[14], [18], [20], [22], [24], [26]–[30] (2,953 cases), and no statistical heterogeneity was observed among various trials (*P* = 0.73, *I* ^2^ = 0%). Therefore, a fixed-effect model was used for the meta-analysis. The results showed no statistically significant difference in the miscarriage rate between the GnRH antagonist group and the GnRH agonist group (OR:1.17,95%CI:0.85∼1.61; *P* = 0.34; Figure S7 in File S1).

#### The cycle cancellation rate

This outcome measure was included in 21 studies [8]–[14], [17]–[23], [25]–[30] (3,823 cases), and no statistical heterogeneity was observed among various trials (*P* = 0.11, *I* ^2^ = 29%). Therefore, a fixed-effect model was used for the meta-analysis. The results showed no statistically significant difference in the cycle cancellation rate between the GnRH antagonist group and the GnRH agonist group (OR:1.11,95%CI:0.90∼1.37; *P* = 0.33; Figure S8 in File S1).

### Sensitivity Analysis and Publication Bias

Excluding the maximum weight studies [9], [21], [27], [28], [30] in the following outcomes, sensitivity analysis showed that there was no statistically significant difference in clinical pregnancy rate between the GnRH antagonist group and the GnRH agonist group. (OR:0.89,95%CI:0.76∼1.04; *P* = 0.14), but the results of HCG endometrial thickness (MD: −0.12, 95%CI: −0.43∼0.19; *P* = 0.44), number of oocytes retrieved (MD: −1.56, 95%CI: −2.05∼−1.07; *P*<0.0001), ongoing pregnancy rate (OR: 0.90, 95%CI: 0.75∼1.09; *P* = 0.29), live birth rate (OR:0.91,95%CI:0.60∼1.36; *P* = 0.63), OHSS rate (OR:0.64,95%CI:0.44∼0.92; *P* = 0.02), miscarriage rate (OR:1.24,95%CI:0.87∼1.76; *P* = 0.24) and cycle cancellation rate(OR:1.00,95%CI:0.80∼1.26; *P* = 0.99)were steady. Begg's funnel plot was symmetrical and there was no notable publication bias (Begg's Test *P* >0.05, Figure S9 in File S1).

## Discussion

The results of this systematic review showed that in IVF-EF patients with supposed normal responses, the number of stimulation days, Gn amount, E2 value on the day of HCG, number of oocytes retrieved, and incidence of OHSS were significantly lower with the GnRH antagonist protocol than with the GnRH agonist long protocol. The endometrial thickness on the day of HCG, ongoing pregnancy rate, live birth rate, miscarriage rate, and cycle cancellation rate were similar in the 2 groups. The difference in clinical pregnancy rate between the two groups was uncertain.

### Definition of patients with supposed normal responses

The ovarian reserve function is the foundation of ovarian responses to COH. Predictive indicators of this function include the basal follicle stimulating hormone (bFSH), anti-Mullerian hormone (AMH), and inhibin B levels, as well as the antral follicle count (AFC). However, no method is currently available to accurately measure ovarian reserve. Ovarian responses can be roughly divided as high response, normal response, and low response. There remain no clear diagnostic criteria for these 3 types of response. Therefore, definitions of the scopes of these 3 responses are vague. In addition, it is difficult to accurately predict ovarian responses using the currently available approach. Patients defined as having normal responses often presented with the 3 above-described response types to COH. Therefore, the definition of a supposed normal response for the patients included in this study is, to some extent, merely an assumption.

### Interpretation of the findings

PCOS, a high AMH level, and a younger age (<35 years) are considered primary risk factors for the occurrence of OHSS; a high blood E2 level during the COH process and an excessive number of follicles on the oocyte retrieved day are considered secondary risk factors for the occurrence of OHSS. Compared with the GnRH agonist protocol, the GnRH antagonist protocol had a shorter stimulation time, lower required Gn amount, lower number of oocytes retrieved, and lower E2 level on the day of HCG, and therefore, the incidence of OHSS was lower in this group.

The patients included in this study were assumed to have normal responses, and the clinical pregnancy rate was significantly lower with the GnRH antagonist protocol than with the GnRH agonist protocol. But the sensitivity analysis showed there was no statistically significant difference in clinical pregnancy rate between the two groups. So it could not conclude that the clinical pregnancy rate of GnRH antagonist protocol was lower than that of GnRH agonist protocol.

Among the patients with low responses, the clinical pregnancy rates were similar in the GnRH antagonist and GnRH agonist groups [4], [31]. Among the PCOS patients, the clinical pregnancy rate of the GnRH antagonist group was similar to that of the GnRH agonist group [4], [32]. A comprehensive analysis of the patients with all response types showed that the clinical pregnancy rate was significantly lower with the GnRH antagonist protocol than with GnRH agonist treatment [2], [4], suggesting that the clinical pregnancy rates could differ between the 2 groups of patients with different response types.

The differences in the ongoing pregnancy and live birth rates between the 2 groups were not statistically significant, suggesting that the final outcome of pregnancy was similar, regardless of whether the GnRH antagonist or GnRH agonist protocol was used.

The differences in the miscarriage and cycle cancellation rates between the 2 groups were not statistically significant, suggesting that these 2 outcome measures were not causes of the difference in the clinical pregnancy rate between the 2 protocols.

The effects of the GnRH antagonist and GnRH agonist protocols on the endometrium have not been clarified [33], [34]. In this study, the difference in endometrial thickness on the day of HCG between the 2 groups was not statistically significant; however, this is insufficient to demonstrate a difference in the impacts of the 2 protocols on the endometrium.

### Comparison with existing reviews

In the 2011 study conducted by Al-Inany [4], 45 RCTs (n = 7511) were included to compare the GnRH antagonist and standard long GnRH agonist protocols. However, the patients included in the study of Al-Inany represented all response types, with no exclusion of studies including patients with PCOS and low responses. The patients included in our study were assumed to have normal ovarian responses. The studies of patients with low response and high response (PCOS), GnRH antagonist in the context of minimal stimulation protocols and oocyte donation cycles were excluded. The other two RCTs [29], [30] after 2011 were included in our study. In addition, the study by Al-Inany did not analyse outcome measures, such as the number of stimulation days, Gn amount, E2 value on the day of HCG, number of oocytes retrieved, and endometrial thickness on the day of HCG.

### Strength and limitations of this study

The studies included in this systematic review and meta-analysis were screened according to strict criteria. The subjects included patients with supposed normal ovarian responses. Baseline consistency among the treated patients was analysed with regard to age, body mass index (BMI), bFSH, and other factors. The test and control groups in the 23 RCTs were comparable, with a consistent baseline. Single-dose or multiple-dose protocols were used in the GnRH antagonist group, and the standard long protocol was used in the GnRH agonist group. The protocols used in the 2 groups were equivalent and consistent. However, explicit randomised and concealment methods and the blinding method were not used in some studies, and the clinical drug protocols were not identical. All of these discrepancies might lead to bias.

### Implications for clinical practice

Regarding efficacy, the overall clinical results obtained with the GnRH antagonist and GnRH agonist standard long protocols were similar, and the GnRH antagonist protocol had the added characteristics of a shorter stimulation time and smaller required amount of Gn. Regarding safety, the incidence of OHSS was significantly lower with the GnRH antagonist protocol than with the GnRH agonist standard long protocol. In general, before determining a clinical treatment plan, the patient's ovarian responsiveness to COH should be considered when developing a personalised treatment regimen based on biological indicators.

### Implications for future research

The difference between the GnRH antagonist protocol and GnRH agonist standard long protocol for patients with the same response type requires further clarification; however, RCTs based on patients with different response types to compare the GnRH antagonist protocol and GnRH agonist standard long protocol are currently lacking. Therefore, a multi-centre RCT with a rigorous design is expected in the future.

## Conclusion

In conclusion, when used during IVF treatment for patients with supposed normal responses, the GnRH antagonist protocol could significantly reduce the incidence of OHSS while yielding similar ongoing pregnancy and live birth rates compared with those of the GnRH agonist standard long protocol. But it was not sure that there was any difference in clinical pregnancy rate between the two groups. To further clarify the differences between the GnRH antagonist protocol and the GnRH agonist standard long protocol for patients with different types of ovarian responses, a multi-centre RCT with a rigorous design is needed in the future.

## Supporting Information

File S1 **Figure S1 in File S1. Risk of bias for each included study. +  =  low risk; ?  =  Unclear; –  =  high risk. Figure S2 in File S1. Forest plot of the comparison of the GnRH antagonist versus GnRH agonist protocol for the number of stimulation days. Figure S3 in File S1. Forest plot of the comparison of the GnRH antagonist versus GnRH agonist protocol for the Gn amount(amp). Figure S4 in File S1. Forest plot of the comparison of the GnRH antagonist versus GnRH agonist protocol for the endometrial thickness on the day of HCG(mm). Figure S5 in File S1. Forest plot of the comparison of the GnRH antagonist versus GnRH agonist protocol for the E2 value on the day of HCG(pg/ml). Figure S6 in File S1. Forest plot of the comparison of the GnRH antagonist versus GnRH agonist protocol for the number of oocytes retrieved. Figure S7 in File S1. Forest plot of the comparison of the GnRH antagonist versus GnRH agonist protocol for the miscarriage rate. Figure S8 in File S1. Forest plot of the comparison of the GnRH antagonist versus GnRH agonist protocol for the cycle cancellation rate. Figure S9 in File S1. Begg's funnel plot of the comparison of the GnRH antagonist group versus GnRH agonist group for the clinical pregnancy rate.**
(RAR)

Checklist S1 (DOC)
